# Transcranial Direct Current Stimulation in Sports Training: Potential Approaches

**DOI:** 10.3389/fnhum.2013.00129

**Published:** 2013-04-09

**Authors:** Michael J. Banissy, Neil G. Muggleton

**Affiliations:** ^1^Department of Psychology, Goldsmiths University of London, London, UK; ^2^Brain and Behavior Laboratory, Institute of Cognitive Neuroscience, National Central University, Jhongli, Taiwan

Successful sporting performance is a result of sustained excellence in a number of factors. Some of these, such as high levels of physical fitness, are common to most sports but it is also true that a large number of sports require excellent levels of motor coordination and less well-defined factors such as “the will to win.” While these factors are most likely to spring to mind when considering excellent sporting performance, a range of other abilities may also contribute to success depending on the nature of the sport. These include visual detection (e.g., when a fast moving object such as a cricket ball must be judged or hit) and anticipation (e.g., in situations where the ability to predict or infer the behavior of an opponent may be beneficial). For example, tennis players show a range of enhanced abilities compared to less skilled players related to anticipation in short time windows, such as extracting information from posture (Rowe and McKenna, 2001) or use of more efficient visual search behaviors. Recently it has also been shown that tennis players show better temporal preparation compared to fitness-matched controls (Wang et al., 2013a) as well as showing altered patterns of inhibitory control (Wang et al., 2013b), implying that they may be better able to modulate their behavior.

There are always gains to be made in performance as a consequence of identifying the essential characteristics that contribute to success in a particular sport and then either identifying individuals that show them or training these skills to higher levels in those already involved in the sport. While it is beyond doubt that many of these factors remain to be clearly identified, the ability to improve some or all of these aspects of performance beyond the level attainable with current methods would clearly lead to better sporting performance. This leads to the question of whether non-invasive brain stimulation techniques, such as transcranial direct current stimulation (tDCS) can be applied beneficially to fitness, skill levels, or both.

Transcranial direct current stimulation is a non-invasive technique that achieves modulation of brain excitability through the application of direct current at low amplitudes *via* electrodes placed over the scalp (see, for example, Nitsche et al., 2003). The result of this stimulation is modulation of excitability of the region of cortex lying directly under the electrode. The direction of the induced change in excitability depends on the polarity of the electrode over the region being stimulated with anodal stimulation being excitatory and cathodal stimulation being inhibitory. Initial studies employing tDCS modulated motor cortex excitability, showing that the modulated cortical excitability can persist after the cessation of the stimulation and that this effect can be modulated by both intensity and duration of delivery (Nitsche and Paulus, 2001).

So, given that the motor cortex can be modulated by tDCS, the first and most obvious question is whether or not this can be used in a manner that benefits sporting performance. Interestingly, the answer seems to be yes for both endurance *and* skill, although there are potential caveats for both cases. It must also be taken into account that the evidence comes from a mixture of studies in patients (such as those who have suffered from a stroke) and healthy participants rather than sports participants.

Neuromuscular fatigue occurs due to both central and peripheral factors (Gandevia, 2001). While peripheral fatigue, associated with the muscles themselves, is a factor people are generally familiar with, central fatigue may also have a significant role to play in sports training. This is characterized by failure of the central nervous system to drive muscles maximally during exercise (Taylor et al., 2006) and is related to changes in spinal motor neurons and to reduced supraspinal drive. Cogiamanian et al. (2007) investigated whether tDCS delivered over motor cortex, which has been demonstrated to be capable of modulating the activity of this region in several studies (e.g., Nitsche and Paulus, 2000), would have any effect on fatigue in normal volunteers. They found that anodal stimulation, which elevates cortical excitability, had effects consistent with a reduction in fatigue in comparison to both no stimulation and cathodal stimulation. The size of the effect was not inconsiderable, with endurance time over 15% longer in the anodal stimulation condition. The findings were argued to be consistent with cortical function having a role in task failure and several possible explanations were proposed such as modulation of supraspinal fatigue or modulation of feedback inhibition systems. The exact mechanism is clearly important when attempting to maximize the benefits of stimulation while minimizing any potential drawbacks, but the main point remains that it is possible to modulate fatigue to a large degree with tDCS stimulation. While the effects seen have a time range of minutes rather than hours following stimulation, it is not hard to see how this may be of potential use in training for a wide range of sports where reduced fatigue during training would be beneficial.

Studies have also investigated the effects of tDCS on motor strength. For example, Tanaka et al. (2009) found that anodal tDCS resulted in a transient increase in maximum leg pinch force in healthy volunteers, similar to effects previously reported for the hand (e.g., Fregni et al., 2005; Hummel et al., 2005, both in patient groups; Boggio et al., 2006 in healthy volunteers), with a duration of around 30 min. A later study (Tanaka et al., 2011) found knee extension force could be similarly modulated, although the duration of the effect was shorter in this case. Notably, it is not always the case that tDCS improves motor strength. For example Cogiamanian et al. (2007) found no modulation of motor strength following tDCS. In this regard, there may be some potential utility in using tDCS as a tool to modulate motor strength, but further work is required to clarify the specific parameters that mediate this effect (e.g., why effects are seen in some, but not all studies).

As well as motor function in terms of fatigue and power, there is also evidence of facilitation of skilled motor performance. Much of this type of investigation has been focused on possible application in rehabilitation in individuals who have suffered from a stroke. For example, Hummel et al. (2005) found that tDCS in stroke patients resulted in improved scores on a test reflecting motor activity related to daily living and that this effect persisted beyond the time of stimulation. It is important in this context to remember the potential complexity of many of the motor behaviors required for what seem like simple daily tasks.

In addition to patient groups, others have highlighted the utility of tDCS in modulating motor learning in typical adults. For example, Stagg et al. (2011) found that for healthy subjects, tDCS during learning of an explicit motor sequence resulted in modulation of performance in a polarity specific manner. Anodal stimulation improved performance and cathodal stimulation reduced learning speed. Interestingly, in this case, delivery of stimulation prior to the task resulted in worse performance whichever polarity was applied. This illustrates the seeming importance of the timing of tDCS stimulation in modulating task performance, acquisition, and consolidation. Similar to the findings of Stagg et al. (2011), Kuo et al. (2008) found disruption to motor learning with anodal tDCS applied before task performance whereas stimulation after training has been reported to result in enhancement (Tecchio et al., 2010). This suggests stimulation prior to task presentation should be avoided if the aim is to benefit performance. Further investigation of the critical window of stimulation would be beneficial. This is also true for investigation of whether there is an interaction with the type of motor learning. The task used by Stagg et al. (2011) was an explicit motor task, which contrasts with Tecchio et al. (2010) but both explicit and implicit motor learning may be of benefit in a range of sports.

It seems therefore, that there is clearly potential for use of tDCS in affecting motor skills related to sporting performance. Retention of such benefits for a sufficiently long duration beyond the stimulation window is also particularly important and there is evidence supporting that this does occur. Reis et al. (2009) reported that anodal tDCS over the motor cortex improved skill acquisition on a difficult motor task, and skill rates remained higher for the stimulation group after 3 months.

Finally, there is increasing documentation of altered (typically enhanced) patterns of behavior beyond the domain of motor skill and fatigue in those involved in high levels of sporting performance. It is therefore possible that these may also be susceptible to beneficial modulation by tDCS but currently there is less information available to determine the plausibility of such an approach or how beneficial it may be for sporting performance. For example, high level tennis players show better performance on a temporal preparation task, but it is to be determined whether it is possible to modulate this performance or if such modulation would improve sporting performance. The first seems highly likely, and there are several examples of improved performance on behavioral tasks with application of tDCS stimulation across a variety of cognitive and perceptual domains (e.g., Cohen Kadosh et al., 2010; Santiesteban et al., 2012; Tseng et al., 2012). For example, Tseng et al. (2012) improved performance of a working memory task using tDCS. However, it is important to note that this effect was seen for the initially poorly performing subjects that were tested with the effect absent for those who initially already performed well. Indeed, limited use has previously been reported for, for example, generalized visual training in sport. For example, Abernethy and Wood (2001) reported little benefit in performance of a racquet sport following such training. However, in the case of tDCS application, it may be that task performance related to an appropriate ability in the sport can be enhanced further and that this change may provide an index of likely benefit, rather than being the same as a potentially more specific training related effect that may not transfer.

In summary, it seems likely that, if suitably employed, tDCS could be of benefit toward improving performance in many sports, either by aiding motor or perceptual learning and/or the effectiveness of training in these domains. However, currently much of the evidence supporting this is theoretical, having been obtained from individuals not involved in a high standard of sport or from patient groups, nor with sporting performance as the measure under consideration. It is also worth noting that it is likely that in a number of sports there are psychological aspects that may contribute to high level performance that are relatively under examined (at least in a sporting context). These, of course, would provide further potential avenues for improved performance.

On a final note, it would be remiss to not consider the ethics of the use of methods such as tDCS to improve sporting performance. While the use of tDCS to provide benefit in individuals with clinical conditions that have significant impact on both their own lives and of their friends and family seems to be entirely reasonable, particularly given the safety levels of the technique, it is by no means clear that this also applies when considering improving performance in healthy normal individuals. While it seems that tDCS may potentially provide training benefits, it is much less clear whether this should be considered as permissible standard practice. However, this is likely to be primarily governed by first, the level of benefit it provides to an individual and second, should it be decided that it is not permitted in athletes, whether it will be possible to regulate it as it seems unlikely that it would ever be possible to determine whether tDCS had been employed in a training regime or not. In the meantime, the potential extent of benefits of tDCS in a sporting environment awaits further clarification.
